# Development and Feasibility Study of a Triage Tool for Early Referral to Spinal Cord Stimulation for Patients With Chronic Low Back and Leg Pain

**DOI:** 10.1002/ejp.4780

**Published:** 2025-01-05

**Authors:** Ferdinand Bastiaens, Miranda L. van Hooff, Ivar J. Bruaset, Els van den Eede, Natasja J. G. Maandag, Erkan Kurt, Monique C. M. Schel‐Huisman, Jessica T. Wegener, Kris C. P. Vissers

**Affiliations:** ^1^ Department of Research Sint Maartenskliniek Nijmegen The Netherlands; ^2^ Department of Anesthesiology, Pain and Palliative Medicine Radboudumc Nijmegen The Netherlands; ^3^ Department of Orthopedic Surgery Radboudumc Nijmegen The Netherlands; ^4^ Anesthesiology Department Sint Maartenskliniek Nijmegen The Netherlands; ^5^ Chronic Pain Department Sint Maartenskliniek Nijmegen The Netherlands; ^6^ Department of Neurosurgery Radboudumc Nijmegen The Netherlands

## Abstract

**Background:**

In recent years, delayed elective care and growing waiting lists increasingly resulted in postponed surgeries for patients with chronic back and leg pain.

**Objective:**

To develop, implement, and evaluate the feasibility of a triage tool for patients with chronic back and/or leg pain to identify those eligible for referral to spinal cord stimulation (SCS) consultation.

**Methods:**

A triage tool was developed, based on Dutch SCS guidelines, literature review and expert panel consultation. The triage process was detected and implemented in collaboration with a multidisciplinary team, prior to first orthopaedic consultation. Feasibility, reliability and predictive accuracy were analysed as part of the evaluation of the triage tool.

**Results:**

The triage indicators included: Pain location (leg/mixed), DN4 > 3, pain duration ≥ 3 months, leg pain ≥ back pain and NPRS leg pain ≥ 5. The triage tool was applied on patients on the orthopaedic waiting list, followed by a full orthopaedic review if they were not excluded. A total of 1025 orthopaedic patients with chronic back and leg pain were assessed with the triage tool. The triage tool was evaluated as feasible (mean System Usability Score 74.2 [SD 11.5]), reliable (inter‐rater reliability [Fleiss' Kappa 0.79], intra‐rater reliability [Cohen's Kappa 0.89]) and accurate (sensitivity [100%], specificity [98.8%], positive predictive value [40%] and negative predictive value [100%]).

**Conclusion:**

Early triage of potential SCS candidates potentially supports rapid and appropriate care allocation, shortens waiting list time and improves clinical outcomes. Future research should explore strategies to optimise the tool's performance in identifying patients most likely to benefit from SCS therapy.

**Significance:**

A novel triage tool was developed to identify patients with chronic back and leg pain for an early referral to SCS. This tool, evaluated for feasibility, reliability, and predictive accuracy, shows promise in reducing waiting times and improving patient selection. It can be a prelude to the further development of decision support for SCS and an acceleration in the care process for SCS candidates.

## 1 Introduction

Recent staff and resource shortages, worsened by the COVID‐19 pandemic, have resulted in the delay of healthcare programs creating long waiting lists for elective care, health loss and increased costs (Johnson et al. 2022; Koushan, Wood, and Greatbanks 2021; Uimonen et al. 2021; Van Ginneken et al. 2022; WHO 2022; Winter, Schreyogg, and Thiel 2020). This was particularly detrimental for patients with chronic low back and leg pain (CLBLP) or persistent spinal pain syndrome type 2, candidates for spinal cord stimulation (SCS). Delays are associated with an increase in pain, pain medication intake and a decline in mental health (Baranidharan et al. 2021; Kumar et al. 2014). This impact of these various causes on healthcare is relevant to the emerging burden on healthcare and highlights the need for efficient, patient‐centred care pathways, emphasising value‐based and digitalised care approaches (Brahmbhatt, Ross, and Moayedi 2022; Lewis 2022; Porter 2010).

Structured triaging and prioritisation methods can effectively reduce waiting times for elective surgery (Rathnayake and Clarke 2021). To optimise the triage process and continuously monitor treatment outcomes for referred patients with CLBLP, an online decision support tool such as the Nijmegen decision‐support tool for chronic low back pain (NDT‐CLBP) in which a biopsychosocial approach is incorporated seems appropriate (van Hooff et al. 2015, 2014). The NDT‐CLBP utilises data from an institutional patient‐based spine outcomes registry and matches patients to suitable treatment modalities through comprehensive web‐based screening questionnaires (van Hooff et al. 2018). Although patients with CLBLP are being referred to the chronic pain department the prognostic profiles for early triage to SCS have not yet been incorporated in the NDT‐CLBP (van Hooff et al. 2018).

There is evidence that SCS can improve quality of life and pain relief. Although, the clinical selection process required to optimise outcomes remains challenging (Bastiaens et al. 2024; Grider et al. 2016; Kapural et al. 2017; Palmer, Guan, and Chai 2019). To optimise these clinical outcomes and cost‐effectiveness of SCS, careful patient selection based on proven predictive factors for successful treatment outcome is essential (Dougherty et al. 2020; McClure et al. 2021). Recent studies have identified predictive factors for successful SCS in patients with CLBLP. A higher functional status was predictive for substantial back pain relief, whereas female sex and longer pain duration were predictive for substantial leg pain relief (Bastiaens et al. 2024). Goudman et al. (2021) developed a prognostic prediction model for holistic responders after SCS. Positive predictive factors included age, back pain, amount of pain medication and functional status (Goudman et al. 2021). Additionally, a consensus study for the appropriate referral and selection of chronic pain patients for SCS, resulted in a list of inclusion and exclusion criteria (Thomson et al. 2020). However, these factors have not been prospectively studied in a heterogeneous population with low back and leg pain, referred to a secondary or tertiary healthcare service.

Under pressure of the COVID pandemic, an initiative was taken to streamline the care pathway for SCS, for patients who are not responsive to orthopaedic treatments or who had no indication for orthopaedic surgery. This initiative included incorporating the NDT‐CLBP and developing an add‐on triage tool for these patients. A triage tool based on predictive indicators for patients with CLBLP will facilitate to identify patients who are optimal SCS candidates and contribute to personalised healthcare (Thomson et al. 2020). The primary objective of this study was to develop, implement and validate a decision‐support tool (‘triage tool’) for patients with chronic back and/or leg pain to identify patients eligible for referral to SCS consultation.

## 2 Methods

### 2.1 Objectives

The primary objective of this study is divided into the following subobjectives:

- Development: Identifying the relevant indicators for triaging patients with chronic back and leg pain for spinal cord stimulation eligibility.
- Implementation: Determine how a triage tool for the identification of patients eligible for referral to spinal cord stimulation consultation could be implemented in an orthopaedic outpatient clinic.
- Evaluation: Determining the feasibility (System Usability Score > 68), reliability (Kappa values ≥ 0.70 for inter‐ and intra‐rater reliability) and predictive accuracy (specificity [Sp] and sensitivity [Se] ≥ 80%) of a triage tool for the identification of patients eligible for referral to spinal cord stimulation consultation (Lewis 2018; Medow and Lucey 2011; Terwee et al. 2007).

### 2.2 Development, Implementation and Evaluation

A mixed methods design was used for this single‐centre study, which was based on a clinical initiative from the orthopaedic spine unit and the chronic pain department of Sint Maartenskliniek Nijmegen, The Netherlands, that started in July 2021.

#### 2.2.1 Development

The development of the triage tool consisted of an iterative process:

1. The standard obligatory criteria for SCS from The Dutch Society of Anesthesiology and the Dutch National Health Care Institute were derived from national guidelines and included in the triage tool (NVA 2020; ZNL 2019).
2. Additional potential indicators, including norm values, were identified through an exploratory review.
3. Subsequently, the selected potential indicators for triage and derived from the exploratory review, were verified by a panel of pain specialists who implant and manage SCS patients, regarding which symptoms are typically present at before implantation in successfully implanted neuropathic pain patients.
4. The triage process and continuous fast‐track for SCS were detected and described in collaboration with a multidisciplinary team of accredited pain specialists, a spine physician, orthopaedic surgeons and healthcare managers, prior to first orthopaedic consultation.

#### 2.2.2 Implementation

The triage process and continuous fast‐track for SCS were implemented prior to orthopaedic consultation and in collaboration with aforementioned multidisciplinary team. The implementation of the prototype triage tool was evaluated in a before‐after design and improved through continuous improvement cycles (Plan‐Do‐Study‐Act [PDSA] cycles). Evaluations were held on a quarterly basis in the first year of implementation with the main researcher and the users of the triage tool. User experiences and preliminary results of the triage were discussed to find possible improvements, resulting in a final triage tool for SCS.

Pain specialists, orthopaedic surgeons and physician assistants performed the triage process. After triage, all the patients were monitored on the received treatment until the end of their therapy at the Sint Maartenskliniek or when they reached a stable situation during their therapy (e.g., continuous periodical care such as a PRF treatment). The final triage tool was evaluated by examining the usability, reliability and predictive accuracy.

#### 2.2.3 Evaluation

To evaluate the feasibility, the users (pain specialists and orthopaedists) were asked to evaluate the usability using the System Usability Scale (SUS Dutch version; Brooke 1996; Ensink, Keijsers, and Groen 2024). The SUS consists of 10 questions with answers given on a five‐point Likert scale from ‘Strongly disagree’ to ‘Strongly agree’. The total score ranges from 0 to 100, with higher scores indicating a higher usability (Brooke 1996; Lewis 2018). Furthermore, a subsample of 50 patients was assessed separately by four clinicians to determine inter‐ and intra‐rater reliability, in accordance with quality criteria for reliability studies (Terwee et al. 2007). The validity of the triage was evaluated by determining the specificity (Sp), sensitivity (Se), negative predictive value (NPV) and positive predictive value (PPV).

### 2.3 Study Population

A convenience sample of 1025 patients on the orthopaedic waiting list was assessed with the triage tool. The orthopaedic waiting list consisted of a heterogeneous population of patients with CLBLP, irrespective of previous spinal surgery, referred from both primary and secondary care settings. The estimated waiting time for an orthopaedic consult ranged from 6 to 12 months. Pain specialists and orthopaedic surgeons, as well as nurse specialists and physician assistants involved in the clinical initiative were involved in the developmental process, continuous improvement cycles and usability examination. The inter‐rater reliability was examined by comparing the triage results of pain specialists, a neurosurgeon and a nurse specialist, whereas the intra‐rater reliability was examined through initial and repeated triage results on similar patients by the same pain specialist. The continuous improvement cycles had a mixed sample of at least 10 members, consisting of pain specialists, nursing specialists (chronic pain department), researchers, orthopaedists and physician assistants.

### 2.4 Data Analysis

Developmental changes in the triage tool and triage process as a result of the evaluation sessions in the PDSA cycles were registered. Descriptive statistics (Mean (SD)) and count (percentages) were used to report triage tool results. The usability analysis consisted of calculating both the total SUS score (< 68 indicating above‐average usability) and the subscores of the individual questions to test them against the standard values (Bangor, Kortum, and Miller 2009; Lewis 2018; Lewis and Sauro 2018). To analyse the inter‐rater and intra‐rater reliability, both Cohen's and Fleiss' Kappa values and the overall, positive and negative agreement were calculated based on the rater's triage conclusion on SCS eligibility (yes/no) (de Vet et al. 2013). Kappa values ≥ 0.70 were considered reliable (Terwee et al. 2007). The predictive accuracy was determined by calculating Se, Sp, negative predictive value and the positive predictive value of the triage tool for SCS indication (including 95% confidence intervals [95%CI]), from a 2 × 2 cross‐table. In this calculation, the rater's triage conclusion is compared to the conclusion on SCS eligibility made in the orthopaedic consult. For a triage tool, a Se and Sp ≥ 80% is expected for it to be considered accurate (Medow and Lucey 2011). All statistical analyses were performed in R studio (version 4.1.2).

### 2.5 Ethics Committee Review and Approval

The local medical ethics committee (METC Oost‐Nederland, the Netherlands) waived ethical approval since the study was not subject to the medical research involving human subjects act (file number: 2022–16016). All the patients referred to the Sint Maartenskliniek in Nijmegen were informed and gave consent for the use of their data for scientific purposes. All data required for triage and scientific analysis were collected from the patients as part of the standard procedure and were pseudonymised for analyses.

## 3 Results

### 3.1 Development of Indicators

In the exploratory review, seven studies and the obligatory criteria for SCS from The Dutch Society of Anesthesiology and the Dutch National Health Care Institute were used to form a list of possible triage indicators (De La Cruz et al. 2015; Deer et al. 2014; Lee and Pilitsis 2006; Nagel, Lempka, and Machado 2014; NVA 2020; Odonkor et al. 2020; Palmer, Guan, and Chai 2019; Thomson et al. 2020; ZNL 2019). After consultation with the involved pain specialists and quarterly PDSA evaluations, six including and seven excluding indicators were selected to define the triage tool. In the quarterly PDSA evaluation, the screening procedure and the implementation of the screening process were established. In addition, the screening criteria were tightened (all indicators must be present) and the DN4 > 3 was added as an indicator. Because the DN4 was a new addition to the outcome register, it was not completed by all patients.

For a positive triage indication to proceed towards positive indication for SCS, all the including indicators should be present. Furthermore, all excluding indicators should be absent. The triage indicators included in the triage tool are shown in Table 1.

**TABLE 1:** Triage indicators (triage tool) for referral to SCS for patients with CLBLP.

Including indicator	Indicative outcome	Excluding indicator	Indicative outcome
Prior back surgery	Yes	Absolute contraindications	No
Pain location	Leg/mixed	Widespread pain	No
Neuropathic pain	DN4 > 3	Alcohol/drugs abuse	No
Pain duration	≥ 3 months	Response PRF/TENS/medication	No
Leg pain ≥ back pain	Yes	Prior SCS treatment	No
NPRS leg ≥ 5	Yes	Anatomic abnormalities	No
		Age ≤ 18 years	No

Abbreviations: DN4, Douleur Neuropathique 4; NPRS, Numeric Pain Rating Scale; PRF, pulsed radio frequency; TENS: transcutaneous electrical nerve stimulation.

### 3.2 Triage Process

The triage process and continuous fast‐track for SCS were detected and implemented in collaboration with a multidisciplinary team, prior to orthopaedic consultation. In the first stage, a pain specialist applied the triage tool based on patient‐reported outcome measures (PROMs) and referral information for patients on the waiting list of the orthopaedic spine unit. In the second stage, an orthopaedic physician reviewed the patients who met the criteria of the triage tool and gave a clinical recommendation. Based on all available information (PROMs, referral letter, specialist reports, radiology) the completeness of the diagnosis and the possibility for surgical treatment was assessed.

**FIGURE 1:**
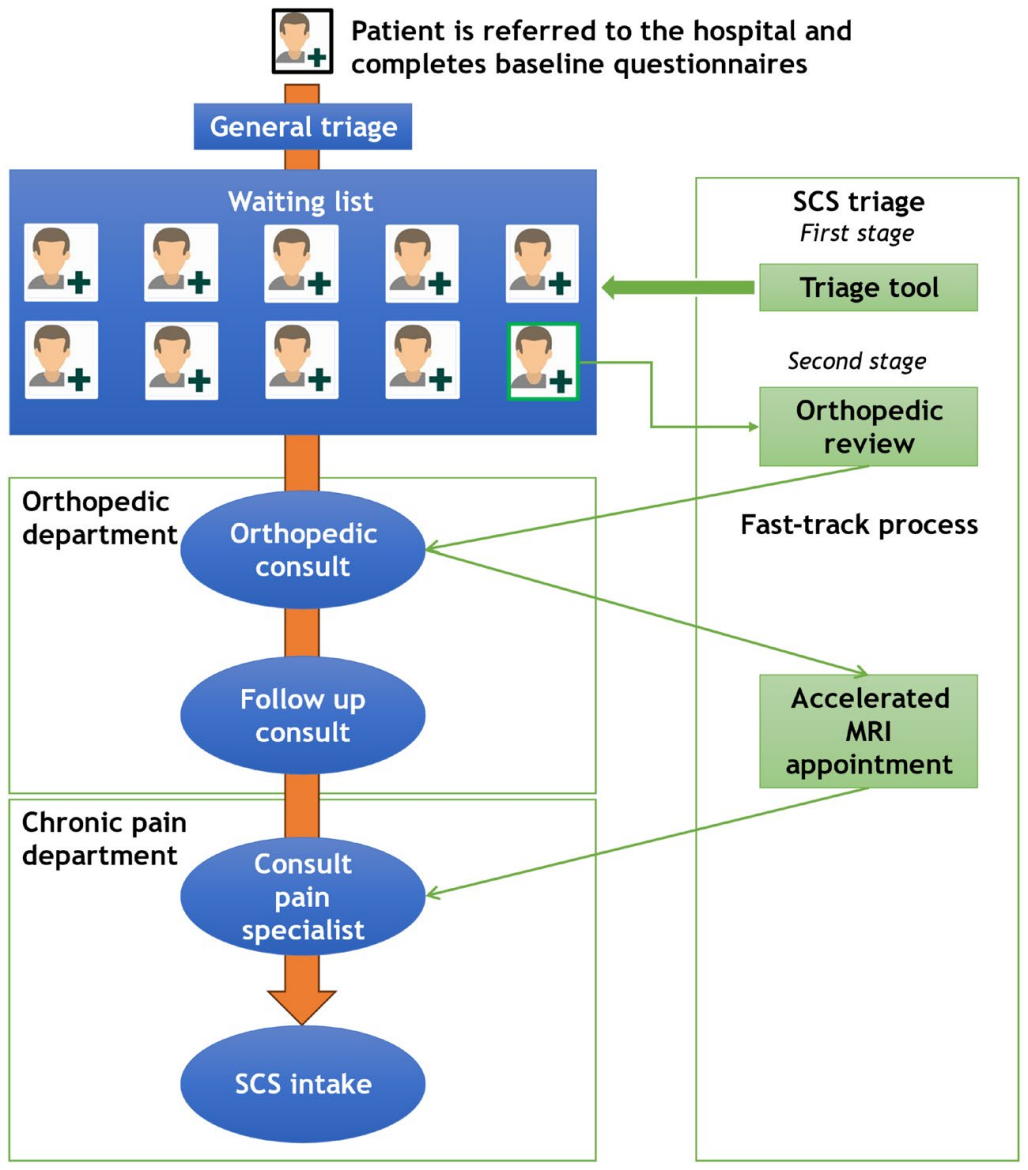
Patient journey from orthopaedic referral towards SCS (marked orange), SCS triage and fast track process (marked green).

When the orthopaedic doctor gave patients a positive triage indication for SCS referral, the patient was included in the fast‐track process: The patient was removed from the waiting list and an appointment was made for an orthopaedic outpatient consultation. Patients who were deemed candidates for SCS after the outpatient consultation were referred to the chronic pain department for an intake, and eventual SCS intake. Those patients also received a fast‐track appointment for an MRI scan. In contrast, patients in the regular patient journey were placed on the waiting list, where the waiting time was determined based on a general orthopaedic triage. In a live consultation, orthopaedic treatment options were explicitly considered first, before other treatment options were considered. An overview of the triage process and fast‐track process is shown in Figure 1.

### 3.3 Evaluation

#### 3.3.1 Reliability

Inter‐rater reliability of the triage tool was classified as ‘good’ with a Fleiss' Kappa of 0.79 overall, with the one‐on‐one Cohen's Kappa ranging from 0.66 to 0.95 (Table 2). Overall agreement was 93%, whereas positive and negative inter‐rater agreement were 95% and 84% respectively. Intra‐rater reliability showed a Cohens Kappa of 0.89 (‘reliable’) between triage ratings, with an overall agreement of 96%. Positive and negative intra‐rater agreement were determined as 97% and 92% respectively.

**TABLE 2:** Interrater reliability per rater (Cohen's Kappa).

	Pain specialist 1	Pain specialist 2	Neurosurgeon	Nursing specialist
Pain specialist 1	X	0.95^a^	0.66	0.84^a^
Pain specialist 2	0.95^a^	X	0.70^a^	0.89^a^
Neurosurgeon	0.66	0.70^a^	X	0.67
Nursing specialist	0.84^a^	0.89^a^	0.67	X

^a^ Reliable, according to the threshold (≥ 0.70).

#### 3.3.2 Usability

The SUS questionnaire was answered by six users of the triage tool (two pain specialists, one nursing specialist (chronic pain department), one spine physician and two physician assistants (orthopaedic spine department)). The mean SUS score for the SCS triage tool was 74.2 (SD 11.5), which is classified as ‘Good’ or ‘B+’ (Bangor, Kortum, and Miller 2009; Lewis 2018). Looking at the subscores on the items of the SUS, all of them achieved their respective benchmark scores, with the exception of item 5 (Lewis and Sauro 2018). Results per item are shown in Table S1. A complete overview of the subscores can be seen in Table 3.

**TABLE 3:** Subscores of the items of the system usability scale.

System usability scale items	Subscore	Benchmark score
1. I think that I would like to use this system frequently	4.17^a^	> 3.39
2. I found the system unnecessarily complex	2.17^a^	< 2.44
3. I thought the system was easy to use	4.17^a^	> 3.67
4. I think that I would need the support of a technical person to be able to use this system	1.83^a^	< 1.85
5. I found the various functions in this system were well integrated	3.50	> 3.55
6. I thought there was too much inconsistency in this system	2.17^a^	< 2.20
7. I would imagine that most people would learn to use this system very quickly	4.17^a^	> 3.71
8. I found the system very cumbersome to use	2.17^a^	< 2.25
9. I felt very confident using the system	3.83^a^	> 3.72
10. I needed to learn a lot of things before I could get going with this system	1.83^a^	< 2.09

^a^ Benchmark score achieved.

#### 3.3.3 Predictive Accuracy

In total, 1025 orthopaedic patients with CLBLP on the waiting list were assessed with the SCS‐triage tool. Of these, 90 patients were identified for the second stage of the triage. After the triage assessment by the orthopaedic physician, 20 patients followed a fast‐track process towards an orthopaedic consultation. Of these 20 patients, eight of them were referred to the chronic pain department as a potential SCS candidate. An overview of the patient characteristics is shown in Table S2.

Based on the combination of the result of the SCS‐triage tool and the indication of the orthopaedic consult on SCS‐eligibility, the SCS‐triage tool had a Se of 100% (95%CI 87.5–1) and a Sp of 98.8% (95%CI 97.7–99.5). The positive predictive value was 40% (95%CI 23.4–59.4), whereas the negative predictive value was 100% (95%CI 99.7–1). A complete overview is shown in Table 4.

**TABLE 4:** Predictive accuracy of the SCS triage tool.

Predictive accuracy	SCS referral	No SCS referral	Total	
Triage indication	8	12	*20*	**PPV 40%**
No Triage indication	0	1005	*1005*	**NPV 100%**
Total	*8*	*1017*	*1025*	
	**Se 100%**	**Sp 98.8%**		

*Note:* Bold indicates the calculated sensitivity, specificity, PPV and NPV and italics indicates the summed‐up total values.

Abbreviations: NPV, negative predictive value; PPV, positive predictive value; Se, sensitivity; Sp, specificity.

## 4 Discussion

In this study, we developed, implemented and evaluated a triage tool for patients with chronic back and/or leg pain to facilitate the identification of patients eligible for referral to SCS consultation. This triage tool was developed based on Dutch SCS standards, a literature search, expert panel consultation. The triage process and continuous fast‐track for SCS referral were implemented in collaboration with pain specialists, orthopaedic surgeons and healthcare managers, prior to orthopaedic consultation and PDSA cycles. In total, 1025 orthopaedic patients were triaged with the tool, of which 20 patients received a positive indication. The triage tool was evaluated as feasible (usability, mean SUS score 74.2 [SD 11.5]), reliable (inter‐rater reliability [Fleiss' Kappa 0.79], intra‐rater reliability [Cohen's Kappa 0.89]) and accurate (Se [100%], Sp [98.8%], positive predictive value [40%] and negative predictive value [100%]) for use in clinical practice, meeting the set standards. This demonstrates that this triage tool is a reliable, accurate, useful and rapid method to identify and allocate potential SCS candidates from a heterogeneous group of patients on an orthopaedic waiting list of a tertiary hospital.

### 4.1 Comparison With Known Literature

Thomson et al. (2020) developed an e‐health tool for referral and selection of chronic pain patients for SCS and was part of the additional literature used in the development triage tool, and shares many similarities with our triage tool (Thomson et al. 2020). However, there are some major differences, for example, our triage tool is focused on patients with back and/or leg pain, where the e‐health tool covers more diverse and multiple indications for SCS (e.g., chronic regional pain syndrome). In addition, our triage tool's set of criteria is developed for use before a consultation has taken place. The application of the e‐health tool also takes place at a later point in the patient journey, which also influences the target population and the available information (Thomson et al. 2023). Looking at the literature on patient selection criteria published after the development of the triage tool, several factors can be identified that are absent in the triage tool developed in our study. These factors are for example, psychological factors (e.g., anxiety or depression), type of pain and trial‐related factors (paresthesia) (Edelbroek et al. 2022; Shanthanna et al. 2023; Thomson et al. 2022). A large part of the triage tool is based on Dutch guidelines and reimbursement rules. Other guidelines (e.g., the National Institute for Health and Clinical Excellence) are in line with the triage tool but are less specific on criteria related to prior back surgery and dominant leg pain (Cruccu et al. 2016; NICE 2008; Centers for Medicare and Medicaid Services 2018).

### 4.2 Strengths and Limitations

This is the first study to focus on the development, implementation, an evaluation of a triage tool for early SCS referral. The strengths of this study lie in its novelty, addressing a previously unexplored area, and its reliance on a robust dataset derived from a large sample size. Furthermore, the triage tool demonstrates practicality, relying solely on PROMs and referral information. This makes it accessible for use and implementation, especially in primary care, and remote areas where access to specialised diagnostic care may be limited. Moreover, by facilitating faster allocation of patients to the appropriate practitioners, the tool has the potential to streamline and shorten appropriate care pathways, potentially reducing fragmentation and enhancing organisational efficiency within healthcare systems (Maruthappu, Hasan, and Zeltner 2015; Misra et al. 2020). In this study, the estimated waiting time of the patients included in the fast track process was cut down by months, excluding the waiting time between the first orthopaedic consult and the continuation of the care pathway. Several limitations warrant consideration. Notably, the low number of positive triage indications provided by the triage tool may skew the positive predictive accuracy, Se and Sp. Future research endeavours should prioritise investigating the reasons behind the low number of positive indications and exploring strategies to optimise the tool's performance in identifying patients who stand to benefit from SCS therapy. In addition, more research is needed to study the effect of the triage tool on both the waiting time of triaged patients, as well as the waiting list in general, preferably in a controlled setting. Furthermore, future research is needed to evaluate the impact of improved patient selection through the triage tool on the long‐term clinical effectiveness of SCS. A possible limitation is confounding by indication, where patients indicated for SCS referral may benefit more from another treatment. This effect is limited in the study by the clinical judgement during both triage and orthopaedic consultation. Nevertheless, it is advisable to further investigate the triage tool in a randomised setting. Furthermore, the study's reliance on a monocentre design, while beneficial for internal validity, limits the generalisability of the findings to other healthcare settings. For example, if you look at the distribution of Cohen's Kappa's in the inter‐rater reliability, the effectiveness of the triage tool may be influenced by the users and their background (pain specialist, neurosurgeon or nurse specialist). External validation in different hospital contexts serving similar patient populations is imperative to establish the broader applicability and effectiveness of the triage tool.

### 4.3 Implications

Our study leveraged available registry data for the development and evaluation of the triage tool. Efforts to establish and maintain comprehensive outcome registers are essential for ongoing monitoring and evaluation of SCS interventions, facilitating evidence‐based decision‐making and quality improvement initiatives (van Hooff et al. 2015, 2018). The triage tool is incorporated as an add‐on to the NDT‐CLBP, allowing patients with CLBLP to be triaged early from the perspective of chronic pain care. It can be a prelude to the further development of decision support for SCS and an acceleration in the care process for SCS candidates. This outcome registry aligns with the low back pain standard outcomes set by the International Consortium for Healthcare Outcome Measurement and meets standards defined by the Dutch Spine Society and Dutch Society of Anesthesiologists (Clement et al. 2015; van Hooff et al. 2015, 2014). Integrating this tool into a broader, multidisciplinary care framework can improve the overall patient journey and support a more holistic approach to patient care (Thomson et al. 2020; van Hooff et al. 2018).

Furthermore, the absence of psychosocial (contra‐)indicators in the triage tool warrants attention. Clinical experts choose to omit psychosocial questions and PROMs. This was due to the complexity surrounding psychosocial factors and the preference for real‐time assessment during consultations. Moreover, a systematic review by Hamm‐Faber et al. (2024) found contradictory outcomes in terms of the impact of psychopathological disorder on the clinical outcome and revision rate of the SCS system (Hamm‐Faber et al. 2024). Nonetheless, there exists an opportunity to expand the triage tool by incorporating PROMs that focus on psychosocial factors and pain characteristics (e.g., HADS, PCS, DN4), thereby enhancing its ability to comprehensively triage patients and optimise treatment pathways (Celestin, Edwards, and Jamison 2009; Markman et al. 2015).

## 5 Conclusion

Early triage of potential SCS candidates could support rapid and appropriate care allocation, shorten waiting list time and potentially improve clinical outcomes. The developed triage tool is characterised as feasible, reliable and accurate for use in clinical practice with high validity. It can be a prelude to the further development of decision support for SCS and an acceleration in the care process for SCS candidates. Future research endeavours should explore strategies to optimise the tool's performance in identifying patients who are likely to benefit from SCS therapy.

## Author Contributions

This study was designed by F.B., M.L.H., J.T.W, N.J.G.M., E.E. and K.C.P.V. The experiments were performed by F.B., I.J.B., E.E., N.J.G.M. and E.K. The data were analysed by F.B., M.L.H, J.T.W. and K.C.P.V., and the results were critically examined by all authors. F.B. had a primary role in preparing the manuscript, which was edited by M.L.H., J.T.W. and K.C.P.V. All authors have approved the final version of the manuscript and agree to be accountable for all aspects of the work.

## Supporting information


Data S1.
