# Supplementary material for: Development and Feasibility Study of a Triage Tool for Early Referral to Spinal Cord Stimulation for Patients With Chronic Low Back and Leg Pain
Source: Eur J Pain. 2025 Jan 5;29(2):e4780. doi: 10.1002/ejp.4780 (PMC11701354; doi:10.1002/ejp.4780)
Supplement: Supplementary file 1 — Data S1. [file EJP-29-0-s001.docx]

Table S1: Results per item of the System Usability Scale.

|  | Strongly disagree  1 | 2 | (N=6)  3 | 4 | Strongly agree  5 |
| --- | --- | --- | --- | --- | --- |
| 1. I think that I would like to use this system frequently. |  |  |  | 5 | 1 |
| 2. I found the system unnecessarily complex. | 1 | 3 | 2 |  |  |
| 3. I thought the system was easy to use. |  |  |  | 5 | 1 |
| 4. I think that I would need the support of a technical person to be able to use this system. | 2 | 3 | 1 |  |  |
| 5. I found the various functions in this system were well integrated. |  |  | 3 | 3 |  |
| 6. I thought there was too much inconsistency in this system. |  | 5 | 1 |  |  |
| 7. I would imagine that most people would learn to use this system very quickly. |  |  |  | 5 | 1 |
| 8. I found the system very cumbersome to use. | 1 | 4 |  | 1 |  |
| 9. I felt very confident using the system. |  |  | 2 | 3 | 1 |
| 10. I needed to learn a lot of things before I could get going with this system. | 2 | 3 | 1 |  |  |

Table S2: Patient characteristics of the triaged sample (n=1025).

| Patient characteristics | Mean ± SD / % |
| --- | --- |
| Age (years) | 55.3 ± 15.3 |
| Sex (females) | 59.0% |
| BMI | 27.2 ± 5.4 |
| Smoking (yes) | 21.1% |
| Prior back surgery (yes) | 36.4% |
| NPRS legs | 5.0 ± 3.2 |
| NPRS back | 7.1± 2.0 |
| Pain duration |  |
| - - <3 months | 3.1% |
| - 3-12 months | 12.3% |
| - 1-2 years | 12.3% |
| - 2-5 years | 21.1% |
| - 6-10 years | 15.4% |
| - >10 years | 35.9% |
| Pain location |  |
| - Legs | 0.7% |
| - Back | 17.9% |
| - Mixed | 81.5% |
| Predominant pain area |  |
| - Legs | 17.1% |
| - Back | 57.4% |
| - Equal | 25.6% |
| ODI | 41.0 ± 16.1 |
| EQ-5D | 0.5 ± 0.3 |
| DN4 back | 2.8 ± 2.2 |
| DN4 leg | 2.8 ± 2.5 |
